# Kiwifruit defense protein, kiwellin (Act d 5) percutaneously sensitizes mouse models through the epidermal application of crude kiwifruit extract

**DOI:** 10.29219/fnr.v65.7610

**Published:** 2021-10-15

**Authors:** Serina Kinugasa, Shota Hidaka, Serina Tanaka, Eri Izumi, Nobuhiro Zaima, Tatsuya Moriyama

**Affiliations:** 1 Department of Applied Biological Chemistry, Graduate School of Agriculture, Kindai University, Nara, Japan; 2 Agricultural Technology and Innovation Research Institute, Kindai University, Nara, Japan

**Keywords:** kiwifruits, allergens, kiwellin, Act d 5, percutaneous sensitization

## Abstract

**Background:**

Kiwifruit is a popular fruit consumed worldwide and is also used as a cosmetic ingredient. However, it is known to cause allergic reactions in humans. Recent studies have suggested an association between food allergy and food allergens entering the body via the skin. However, percutaneously sensitizing kiwifruit allergens have not been identified in human studies or in animal models.

**Objective:**

This study aimed to identify kiwifruit proteins that percutaneously sensitized mice through the epidermal application of crude extracts from green and gold kiwifruit on the dorsal skin, and serum IgE and IgG1 levels were used as sensitization markers.

**Design:**

BALB/c mice were back-shaved and their skin was exposed to crude extracts from green and gold kiwifruit that contained sodium dodecyl sulfate. Specific IgE and IgG1 antibodies generated and secreted in response to antigens were measured using enzyme-linked immunosorbent assay or immunoblotting.

**Results:**

Skin exposure to kiwifruit extract induced an increase in the levels of kiwifruit-specific IgE and IgG1, which are helper T cell 2-related allergenic antibodies in mice. These antibodies reacted with 18, 23, and 24 kDa proteins found in both green and gold kiwifruits. Thus, three percutaneously sensitizing allergens were identified and purified. Their amino acid sequences partially matched with that of kiwellin (Act d 5).

**Discussion and conclusion:**

Kiwellin has been identified as a plant defense-related protein. Interestingly, many plant allergens are biodefense-related proteins belonging to the pathogenesis-related protein family. Kiwellin, which was discovered to be a transdermal sensitizing antigen, might also be categorized as a biodefense-related protein. This study is the first to identify kiwellin (Act d 5) as a percutaneously sensitizing kiwifruit allergen in a mouse model.

## Popular scientific summary

- In a mouse model, specific IgE and IgG1 antibodies were produced by percutaneous application of crude extracts from green and gold kiwifruit.
- These antibodies reacted with 18, 23, and 24 kDa proteins in both green and gold kiwifruits.
- The amino acid sequences of these three novel proteins partially matched with that of kiwellin (Act d 5), indicating that they could be percutaneously sensitizing kiwifruit allergens.

The prevalence of food allergies that disrupt the quality of life is increasing. Food allergies affect almost 5% adults and 8% children in developed countries (1). The gastrointestinal tract is equipped with an immune system that uses oral tolerance to prevent allergic reactions to food proteins (2). However, abnormal functioning of this tolerance mechanism results in food allergy. In IgE-mediated food allergies, food allergens entering the body stimulate the immune system to produce allergen-specific IgE that binds to Fcε receptors on mast cells, leading to sensitization (3).

A recent study reported an association between food allergy and food allergens entering through the skin (4). Hydrolyzed wheat protein-containing soaps have been reported to induce wheat allergy (anaphylaxis) (5). Percutaneous application of soybean can also induce food allergies (6). Another study reported food allergies that preceded contact urticaria in response to contact with foods such as rice, wheat, fruits, vegetables, fish, shrimp, and cattle fish (7). Thus, many cases of food allergy occur because of the percutaneous entry of food allergens, followed by sensitization. Damage to the barrier function of the skin and inflammation cause percutaneous sensitization (8).

Previously, we identified that principal soybean allergens Gly m 5 and Gly m 6 trigger percutaneous sensitization in a mouse model (9). Using the same model, the soybean trypsin inhibitor Gly m TI was also identified as a soybean allergen that causes percutaneous sensitization (10). These food allergens are also the primary soybean allergens in humans, suggesting that a mouse model can be effectively used for the identification of allergens that cause percutaneous sensitization in humans.

Kiwifruit is rich in vitamin C, polyphenols (11), and dietary fiber, and has also been suggested to be effective in preventing heart disease and cancer (12). Green kiwifruit appeared in New Zealand in 1904, in the United States in 1962, and then in European countries (13). Gold kiwifruit appeared in the international market in 1999 (14). Thus, kiwifruit is a popular fruit consumed globally. However, kiwifruit is associated with high risk of allergenicity that was first reported in 1981 (15). Green and gold kiwifruits exhibit cross-reactivity. At present, 13 allergens have been identified in green kiwifruit and three allergens in gold kiwifruit. Among them, thiol protease 30 kDa actinidin (Act d 1), 24 kDa thaumatin-like protein (Act d 2), 17 kDa PR-10 (Act d 8), 17 kDa MLP/RRP (Act d 11), and 50 kDa 11S globulin (Act d 12) are the major allergens in green kiwifruit. In gold kiwifruit, kiwellin (Act d 5) of 28 kDa has been identified to be the main allergen (16). Kiwifruit can cause a variety of allergic symptoms ranging from mild oral allergy syndrome to serious life-threatening anaphylaxis (17). Kiwifruit allergy may be complicated by birch pollinosis and latex allergy (18). Kiwifruit may also be cross-reactive with other foods such as avocado (19), banana (20), and peanut (21). Thus, kiwifruit can cause various allergic symptoms and is known to contain many types of allergens. The diverse symptoms might develop because of the involvement of diverse sensitization pathways. However, the association between these individual allergen components and sensitization pathways, especially percutaneous sensitization, is unclear.

Therefore, in this study, we aimed to identify kiwifruit proteins that percutaneously sensitize mice, for which we applied crude kiwifruit extract epidermally to the dorsal skin of mice and used serum specific immunoglobulin E (IgE) and immunoglobulin G1 (IgG1) as sensitization markers.

## Materials and methods

### Materials

Enzyme-linked immunosorbent assay (ELISA) plates were purchased from AGC (Tokyo, Japan). Horseradish peroxidase (HRP)-conjugated goat anti-mouse IgE antibody was purchased from Southern Biotech (Birmingham, AL, USA). HRP-conjugated goat anti-mouse IgG1 antibody was purchased from Bethyl Laboratories (Montgomery, TX, USA). 3,3,5,5-Tetramethylbenzidine (TMB) peroxidase substrate was purchased from Kirkegaard & Perry Laboratories (KPL; Gaithersburg, MD, USA). Coomassie brilliant blue (CBB) R-250, Bradford protein assay reagent, and pentobarbital sodium salt were purchased from Nacalai Tesque (Kyoto, Japan). Enhanced chemiluminescence (ECL) western blotting substrates and X-ray films (Amersham Hyperfilm MP) were purchased from GE Healthcare (Chalfont St. Giles, UK). Luminata Crescendo Western HRP Substrate was purchased from Merck Millipore (Burlington, MA, USA). Immunoreactive enhancers, Can Get Signal solutions 1 and 2, were purchased from TOYOBO (Osaka, Japan). All other chemicals used in this study were of the highest purity available.

### Preparation of kiwifruit extract and determination of protein content

Green and gold kiwifruits were purchased from a supermarket near the university. They were homogenized using a commercial food mixer and extracted through four layers of gauze. The protein content of the obtained extracts was determined using the Bradford method (22) using protein assay CBB solution (Nacalai Tesque, Kyoto, Japan), and the absorbance was measured at 595 nm.

### Animal studies

Six-week-old female BALB/c mice were purchased from Japan SLC Inc. (Shizuoka, Japan) and used for percutaneous sensitization treatment, as shown in Fig. 2. The mice were fed a commercial chow without kiwifruit proteins (MF, Oriental Yeast, Tokyo, Japan) and water was provided *ad libitum*. The mice were acclimated for 7 days before the start of the experiment. All animal experiments were approved by the Kindai University Animal Experiment Commission (Approval No. KAAG-26-004).

### Percutaneous sensitization

After shaving the occipital region of the mice with an electric shaver under anesthetic conditions, the remaining hair was removed using a hair removal cream (Veet, Reckitt Benckiser, Berkshire, UK), and tape stripping was performed 10 times. A mixture of midazolam (Astellas Pharma, Tokyo, Japan), butorphanol (Meiji Pharmaceutical, Tokyo, Japan), and medetomidine (Nippon Zenyaku Kogyo, Fukushima, Japan) was used for giving anesthesia. The treatment was performed weekly. Transdermal sensitization was induced by applying respective samples to the skin after treatment. Three groups such as control, green kiwifruit, and gold kiwifruit were established (*n* = 5 or 6).

The control group received 5% sodium dodecyl sulfate (SDS) alone, the green kiwifruit group received 4 mg/mL of green kiwifruit extract in 5% SDS, and the gold kiwifruit group received 4 mg/mL of gold kiwifruit extract in 5% SDS. Four times a week for a total of 5 weeks, 50 μL of the sample was applied to the epidermis using a micropipette. Then, the serum was collected weekly. At 5 weeks, all the mice were anesthetized by injecting sodium pentobarbital salt intraperitoneally. The mice were then sacrificed by cervical dislocation. The mice were weighed weekly to determine whether their growth was inhibited.

### Enzyme-linked immunosorbent assay

IgE and IgG1 levels in the sera of mice were measured by performing ELISA. Green and gold kiwifruit extracts (20 μg/mL each) in phosphate-buffered saline (PBS) were added to the ELISA plate (AGC: Tokyo, Japan) and solidified overnight at 4°C. The plates were blocked with 1% BSA in 0.1% Tween 20 (PBST) for 1 h at room temperature and washed three times with 100 μL of PBST; 50 μL of each of the sera diluted with 1% BSA was added and incubated at 37°C for 1 h. After washing five times with PBST, 50 μL of secondary antibodies were added respectively, and incubated at 37°C for 1 h. After washing five times with PBST, the bound secondary antibody was detected by reacting with 50 μL of TMB-peroxidase substrate (KPL, Gaithersburg, MD, USA). The response was stopped by adding 50 μL of 1 M phosphate, and the signal was amplified. Sera were diluted 100-fold for IgE measurements and 5,000-fold for measuring IgG1 levels. For secondary antibodies, 8,000-fold diluted goat anti-mouse HRP-conjugated IgE (Southern Biotech: Birmingham, USA) and 1% BSA were used to measure IgE levels, whereas 50,000-fold diluted goat anti-mouse IgG1 HRP-conjugated antibody (Bethyl Laboratories, Montgomery, TX, USA) and 1% BSA were used to measure IgG1 levels. The absorbance of each well was measured at 450 nm using a plate reader (Wallac ARVO SX 1420 multilabel counter, PerkinElmer, Waltham, MA, USA). The absorbance of two wells was measured for each reading, and their means were used for statistical analysis.

### Electrophoresis and immunoblotting

Green kiwifruit and gold kiwifruit extracts were separated by sodium dodecyl sulfate-polyacrylamide gel electrophoresis (SDS-PAGE) (23) by running at 200 V (constant voltage) for approximately 35 min. For the confirmation of protein patterns, SDS-PAGE gels were stained with CBB. After SDS-PAGE, the gels were transferred to polyvinylidene difluoride (PVDF) membranes for immunoblotting (Immobilon-P; Millipore, Billerica, MA, USA) in a semi-dry manner at 15 V (constant voltage) for 30 min (24).

Blocking was performed by immersing the PVDF membrane in 5% skim milk solutions dissolved in PBST for 1 h. For IgE measurement, the membrane was washed two times with PBST for 5 min. The serum diluted 100-fold using Can Get Signal2 (Toyobo: Osaka, Japan) and the serum diluted 200-fold using skim milk solution were reacted overnight at 4°C for IgG1 measurement. After three 10-min washes with PBST, IgE was measured using an HRP-conjugated rat monoclonal [23G3] anti-mouse IgE antibody (Abcam: Cambridge, UK) that was diluted 4,000-fold by Can Get Signal2 and reacted with the protein surface of the PVDF membrane for 1 h. IgG1 was measured using an HRP-conjugated goat anti-mouse IgG1 antibody diluted 50,000-fold with skim milk solution. Four 10-min washes with PBST were followed by reaction with Luminata Crescendo Western HRP Substrate (Millipore Corporation: Billerica MA01821, USA for IgE determination) and Amersham ECL Western Blotting Detection Reagents (GE Healthcare: Chalfont St for IgG1 determination. Giles, UKs) for 1 min, and luminescence signals were detected using X-ray films (Amersham Hyperfilm MP; GE Healthcare).

In addition, the presence of actinidin was confirmed by immunoblotting. After performing SDS-PAGE, blotting, and blocking as described previously, an anti-actinidin antibody diluted five-fold with skim milk was reacted for 1 h. After washing four times for 10 min with PBST and with Amersham ECL Western Blotting Detection Reagents (GE Healthcare) for 1 min, the emission signal was detected using X-ray films (Amersham Hyperfilm MP; GE Healthcare). The actinidin antibody used was obtained from the allergen eye ELISA II kiwifruit kit (Prima Meat Packers, Ltd., Ibaraki, Japan).

### Purification and identification of kiwifruit antigens

IgG1 binding proteins were purified by ammonium sulfate precipitation, followed by ion-exchange and gel-filtration chromatography to identify percutaneously sensitizing antigens in green kiwifruit. In every purification step, SDS-PAGE and immunoblotting were performed. IgG1-binding proteins were detected using mixed mouse sera. The sera of the mice used were mixed with equal amounts of serum from six green kiwifruit-applied mice collected at week 4 and used as primary antibodies.

### Ammonium sulfate precipitate

Green kiwifruit extract (100 mL) in buffer A (10 mM Tris-HCl, pH 7.5, 1 mM EDTA) containing the protease inhibitor mix (Nacalai Tesque) was freshly prepared for purification. Ammonium sulfate was added stepwise to the above solution, and precipitate was obtained by centrifugation at each step. Samples precipitated with ammonium sulfate (0–20%, 20–40%, and 40–60%) were recovered and resolved with 10 mL of buffer A.

### Ion-exchange chromatography

The samples obtained after ammonium sulfate precipitation were desalted using a PD-10 mini-column (GE Healthcare), and then manually prepared ion-exchange chromatography was conducted using Super Q anion-exchange gels (TOSOH, Tokyo, Japan) (bed volume: 5 mL) to separate proteins in a stepwise manner using NaCl.

### Gel-filtration chromatography

The samples obtained by ion-exchange chromatography were concentrated using Amicon Ultra-15 10 K filters (Merck Millipore, Burlington, MA, USA) and gel-filtration chromatography (TOSOH G3000SW) connected to high-pressure liquid chromatography (HPLC) (Hitachi L-6200) for further purification (flow rate: 0.5 mL/min). Elution fractions (0.5 mL/fraction) from gel-filtration chromatography were used to perform CBB staining and immunoblotting after resolving the proteins by SDS-PAGE as described earlier.

### N-terminal amino acid sequence analysis

The sample obtained from gel-filtration chromatography was subject to SDS-PAGE and transferred onto a PVDF membrane as described earlier. The PVDF membrane was stained with Ponceau S, and the band corresponding to the IgG1 binding protein was cut and collected. The N-terminal amino acid sequence of the sensitizing antigen was determined using Edman degradation (25) (analyzed at Hokkaido System Science, Sapporo, Japan).

## Statistical analysis

Statistical differences were determined using Tukey’s multiple comparison test and Dunnett’s test. A *P* < 0.05 was considered statistically significant. Statistical analysis was performed using the StatView 5.0 software (SAS Institute, Cary, NC, USA).

## Results

### Protein patterns of green kiwifruit and gold kiwifruit

**Fig. 1:**
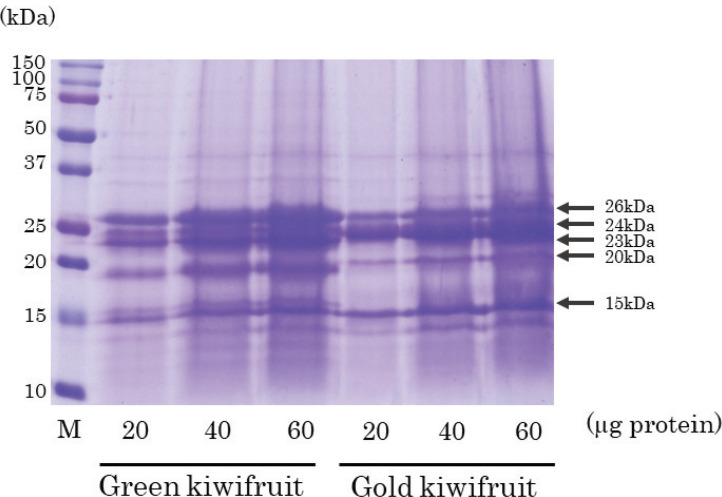
Coomassie brilliant blue staining of green kiwifruit and gold kiwifruit proteins. Green kiwifruit and gold kiwifruit proteins (20, 40, 60 μg/lane) were subject to sodium dodecyl sulfate-polyacrylamide gel electrophoresis and stained with Coomassie brilliant blue. Detailed information is given in ‘Materials and methods’.

The protein content in green kiwifruit and gold kiwifruit was confirmed by CBB staining after SDS-PAGE, and the patterns of protein bands between the two species were generally similar, although there were slight differences (Fig. 1). The major protein bands in these two species included 26, 24, 23, 20, and 15 kDa bands.

**Fig. 2:**
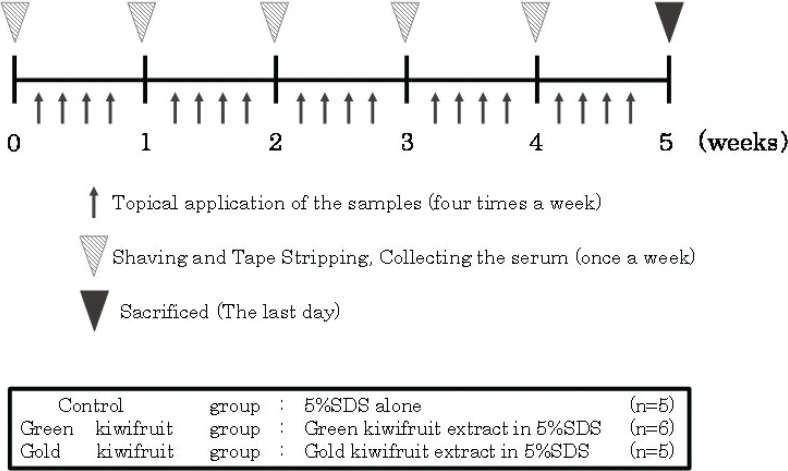
Schema of the percutaneous sensitization protocol. Detailed information is given in ‘Materials and methods’.

### Detection of changes in kiwifruit-specific IgE and IgG1 antibodies determined using ELISA

The experimental animal scheme is summarized in Fig. 2. Body weights were measured weekly to ensure that the mice were not under excessive stress by the application of allergens. Thus, body weights gradually increased normally over time. In addition, no statistically significant difference was observed in body weight changes between the groups, and hence, the mice were raised normally and no differences in growth were observed depending on the difference in the applied samples (data not shown).

**Fig. 3:**
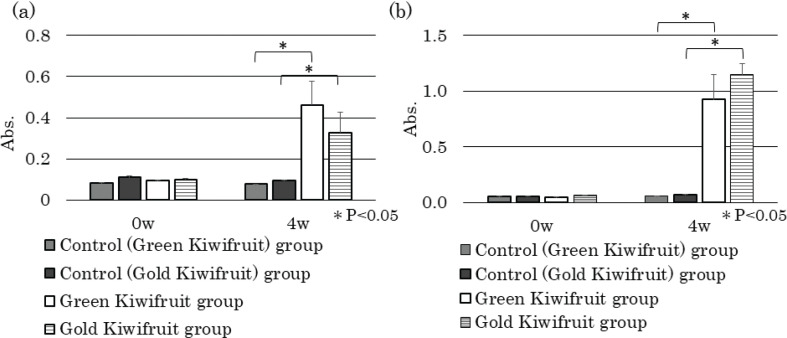
Effect of percutaneous sensitization with kiwifruit extracts on kiwifruit-specific antibody levels in mice sera. Kiwifruit protein-specific IgE levels (a) and IgG1 levels (b) in control and kiwifruit groups at 0 and 4 weeks were determined using ELISA performed with each kiwifruit protein coated plates. Sera from the control group were measured two-way, one by using green kiwifruit as a solid-phase (Control (Green Kiwifruit)) and the other by using gold kiwifruit as a solid-phase (Control (Gold Kiwifruit)). Absorbance data are expressed as means ± standard deviations; control group (*n* = 5), green kiwifruit group (*n* = 6), gold kiwifruit group (*n* = 5). *P < 0.05.

Sera from weeks 0 and 4 were used to assess antigen-specific IgE and IgG1 in sera by performing antigen solid-phase ELISA. Specific IgE (Fig. 3a) and IgG1 (Fig. 3b) levels were significantly higher in both green kiwifruit and gold kiwifruit groups compared with the control group (*P* < 0.05). Sera from the control group were measured in two ways, one by using green kiwifruit as a solid-phase (green control) and the other by using gold kiwifruit as a solid-phase (gold control). No significant difference was observed in these values (Fig. 3a and 3b).

### Detection of IgE- and IgG1-binding kiwifruit proteins using immunoblotting

**Fig. 4:**
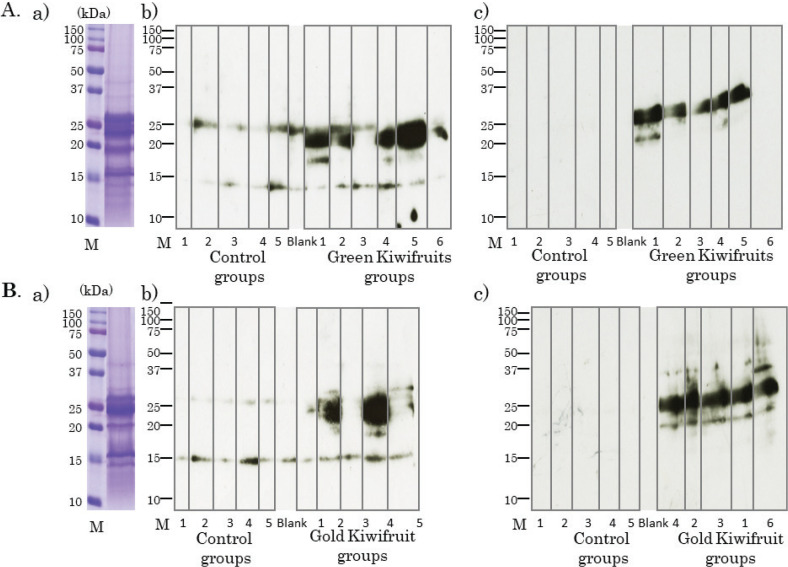
Detection of IgE and IgG1, binding to two species of kiwifruit proteins using immunoblotting. (A) Coomassie brilliant blue staining of green kiwifruit proteins (a). The numbers indicate the individual mice in the control and green kiwifruit groups. Immunoblotting using green kiwifruit proteins, individual mice sera and horseradish peroxidase (HRP)-labeled anti-mouse IgE antibody (b). Immunoblotting using green kiwifruit proteins, individual mice sera, and HRP-labeled anti-mouse IgG1 antibody (c). (B) The numbers indicate the individual mice in control and gold kiwifruit groups. Coomassie brilliant blue staining of gold kiwifruit proteins (a). Immunoblotting using gold kiwifruit proteins, individual mice sera, and HRP-labeled anti-mouse IgE antibody (b). Immunoblotting using gold kiwifruit proteins, individual mice sera, and HRP-labeled anti-mouse IgG1 antibody (c).

To determine which proteins in kiwifruits are bound by IgE and IgG1 antibodies produced in the serum of mice, immunoblots were performed with serum of 4 weeks. Green (Fig. 4A) and gold (Fig. 4B) kiwifruit proteins were run separately and used for immunoblotting. Immunoblots for green kiwifruit proteins revealed multiple antigen-specific IgE (Fig. 4A(b)) and IgG1 bands in the green kiwifruit group (Fig. 4A(c)). Similarly, immunoblots for gold kiwifruit proteins revealed multiple antigen-specific IgE (Fig. 4B(b)) and IgG1 bands in the gold kiwifruit group (Fig. 4B(c)). In the case of IgE, bands reactive to 26 kDa and 15 kDa were also detected in sera from the control groups (Fig. 4A(b) and Fig. 4B(b)). These bands appeared to represent non-specific binding proteins, probably the second antibody directly binding to kiwifruit proteins. In addition, a dense band in the region of 20–25 kDa was observed, and was detected in both the kiwifruit groups (Fig. 4A(b) and Fig. 4B(b)). In the case of IgG1, non-specific binding proteins were not observed, and at least two dense IgG1 bands were detected in the 20–25 kDa region in both the kiwifruit groups (Fig. 4A(c) and Fig. 4B(c)). IgE and IgG1 bands were also detected in the region of 18–20 kDa in sera from several individual mice.

In case of both IgE and IgG1, strong antibody-binding protein(s) in the region of 20–25 kDa were commonly detected, specifically in sera from both the kiwifruit groups, suggesting that both IgE and IgG1 bound to the same protein(s). These proteins were considered to be transdermal sensitizing antigen candidates from both the kiwifruits in this mouse model system.

### Confirmation of cross-reactions between two species of kiwifruit

**Fig. 5:**
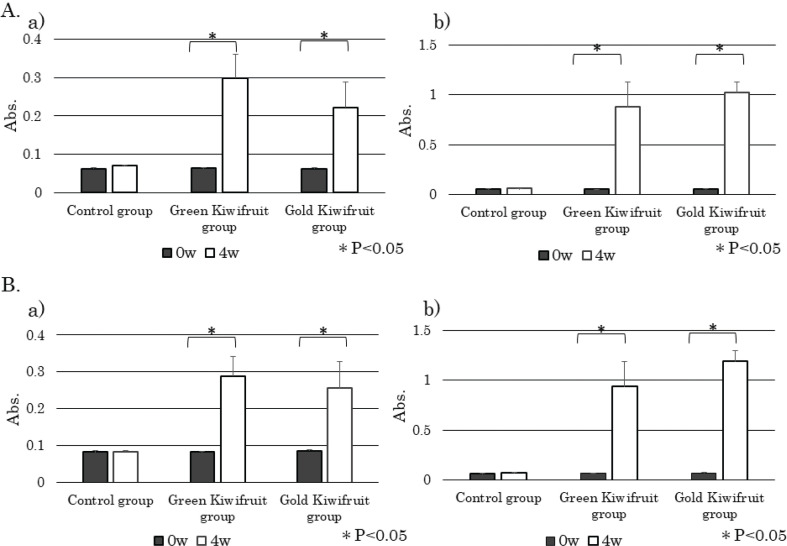
Confirmation of cross-reactions between two species of kiwifruit. (A) The serum IgE (a) and IgG1 levels (b) in control and kiwifruit groups at 0 and 4 weeks were determined using enzyme-linked immunosorbent assay (ELISA) performed with green kiwifruit protein-coated plates. (B) Serum IgE levels (a) and IgG1 levels (b) in control and kiwifruit groups at 0 and 4 weeks were determined using ELISA performed with gold kiwifruit protein-coated plates. Absorbance data are expressed as means ± standard deviations; control group (*n* = 5), green kiwifruit group (*n* = 6), gold kiwifruit group (*n* = 5). *P < 0.05.

**Fig. 6:**
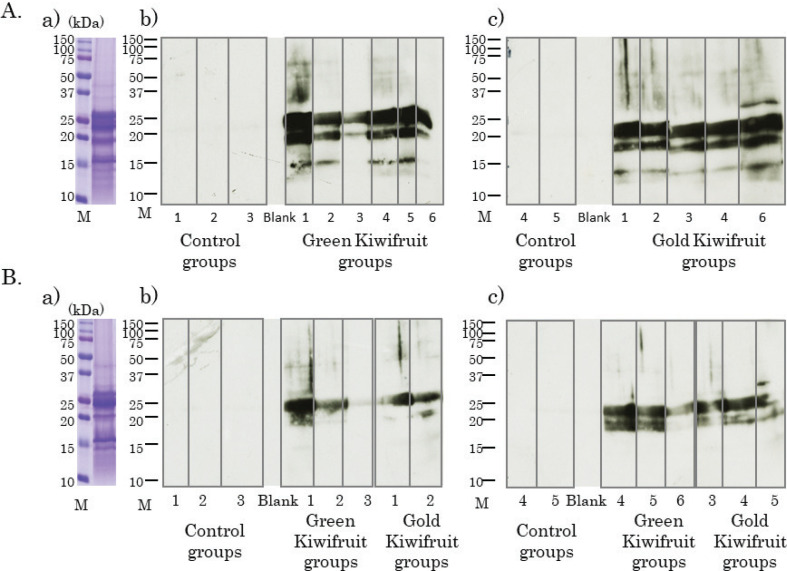
Confirmation of cross-reactions between two species of kiwifruit using immunoblotting. (A) Coomassie brilliant blue staining of green kiwifruit proteins (a). The numbers indicate the individual mice in control and each of kiwifruit groups. Immunoblotting using green kiwifruit proteins, individual mice sera, and horseradish peroxidase (HRP)-labeled anti-mouse IgG1 antibody (b, c). (B) The numbers indicate the individual mice in control and each of kiwifruit groups. Coomassie brilliant blue staining of gold kiwifruit proteins (a). Immunoblotting using gold kiwifruit proteins, individual mice sera, and HRP-labeled anti-mouse IgG1 antibody (b, c).

To confirm the cross-reactions between two species of kiwifruit, we investigated whether IgE and IgG1 antibodies in mice sera applied with each kiwifruit cultivar react similarly to the other kiwifruit cultivar proteins. ELISA analysis showed that serum IgE (Fig. 5A(a)) and IgG1(Fig. 5A(b)) from the green kiwifruit group responded almost equally to green and gold kiwifruit proteins. Serum IgE (Fig. 5B(a)) and IgG1 (Fig. 5B(b)) from the gold kiwifruit group also reacted almost equally to green and gold kiwifruit proteins. Similarly, cross-reactive antibody-binding proteins that appeared to be almost identical were found in each cultivar in immunoblot analyses (Fig. 6). Therefore, the antibody produced for green kiwifruit in this model system cross-reacted with the allergen molecules in the gold kiwifruit proteins, and vice versa (Fig. 6).

### Purification of IgG1-binding proteins from green kiwifruit

As the kiwifruit protein bands (approximately 20–25 kDa) that bind IgE and IgG1 were found to be the same, more sensitive IgG1 was selected for the subsequent purification and identification of sensitizing antigens.

**Fig. 7:**
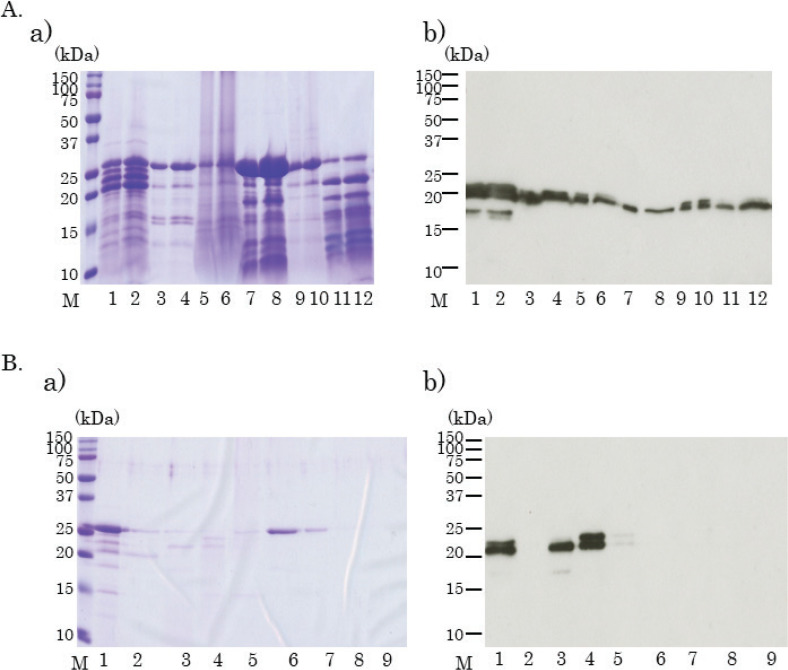
The fraction of kiwifruit proteins using ammonium sulfate precipitation and ion-exchange chromatography. Coomassie brilliant blue staining of fractionated kiwifruit proteins (A(a), B(a)). Immunoblotting using fractionated kiwifruit proteins, mixed mice sera, and horseradish peroxidase-labeled anti-mouse IgG1 antibody (A(b), B(b)). (A) Lanes 1, 3, 5, 7, 9, and 11 were administered 5 μL of the sample. Lanes 2, 4, 6, 8, 10, and 12 were administered 10 μL of the sample. Lane 1~2: the crude extracted sample; Lane 3~4: fractionated samples with 0~20%; Lanes 7~8: fractionated samples with 20~40%; Lanes 11~12: fractionated samples with 40~60% of ammonium sulfate precipitations. Lane 5~6: precipitations of fractionated samples with 0~20% of ammonium sulfate; Lane 9~10: precipitations of fractionated samples with 20~40% of ammonium sulfate precipitations. (B) Lane 1: fractionated sample with 0~20% ammonium sulfate precipitation; lane 2: flow-through fraction from ion-exchange chromatography; lanes 3~9: sample eluted with buffer A containing 0.05 M (3), 0.1 M (4), 0.2 M (5), 0.3 M (6), 0.4 M (7), 0.5 M (8), and 1.0 M (9) of NaCl.

Green kiwifruit extract was fractionated using ammonium sulfate. CBB staining (Fig. 7A(a)) and immunoblotting (Fig. 7A(b)) of each sample performed after ammonium sulfate precipitation revealed that the 20–25 kDa IgG1-binding protein bands were most abundant in the samples precipitated with 0–20% ammonium sulfate. The sample precipitated with 0–20% ammonium sulfate was collected and desalted, and anion-exchange chromatography was performed.

CBB staining and immunoblotting (Fig. 7B) of the ion-exchange chromatography fractions revealed that the proteins in the region of 20–25 kDa were present in the fraction eluted with the buffer containing 0.05 M and 0.1 M NaCl. These two fractions were separately used for subsequent gel-filtration chromatography.

**Fig. 8:**
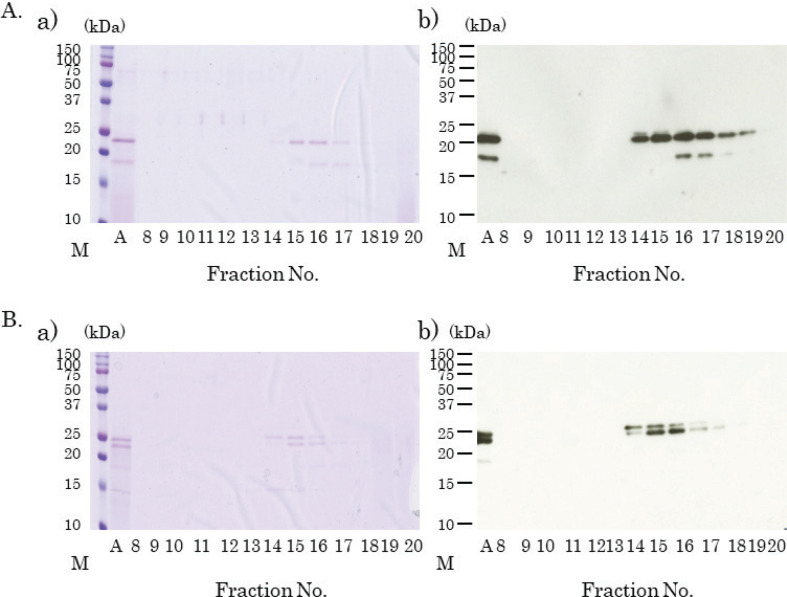
Separation of kiwifruit proteins using gel-filtration high-pressure liquid chromatography (HPLC). Coomassie brilliant blue staining of separated kiwifruit proteins (A(a), B(a)). Immunoblotting using fractionated kiwifruit proteins, mice sera, and horseradish peroxidase-labeled anti-mouse IgG1 antibody (A(b), B(b)). (A) Ion-exchanged 0.05 M NaCl eluted samples with 0–20% ammonium sulfate precipitation, were collected and fractionated on gel-filtration HPLC columns after concentration. Lane A: concentrated sample; Lanes 8~20: eluted fraction number, respectively. (B) Ion-exchanged 0.1 M NaCl eluted samples with 0–20% ammonium sulfate precipitation were collected and fractionated on gel-filtration HPLC columns after concentration. Lane A: concentrated sample; Lanes 8~20: eluted fraction number, respectively.

The samples showing a single band and subject to ion-exchange chromatography with 0.05 M NaCl were collected and further subject to gel-filtration HPLC using G3000SW gel-filtration HPLC columns after concentration. A major IgG1-binding protein band with a molecular weight of approximately 23 kDa was identified in fraction numbers 14–19 (Fig. 8A). A minor IgG1-binding protein band of 18 kDa was also detected in fractions numbers 16 and 17 (Fig. 8A).

The samples subject to ion-exchange chromatography with 0.1 M NaCl were similarly fractionated using gel-filtration columns, and two IgG1 binding protein bands (23 and 24 kDa) were confirmed in the fraction numbers 14–17 (Fig. 8B).

### N-terminal amino acid sequence analysis of green kiwifruit-derived IgG1 binding proteins (18, 23, and 24 kDa)

**Fig. 9:**
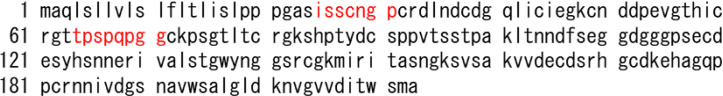
Amino acid sequences of Kiwellin. The N-terminal amino acid sequences identified via Edman degradation are shown in red (GeneBank Protein accession no. AGC39167).

Fractions eluted from gel-filtration columns were transferred to PVDF membranes, and Edman degradation analysis was performed to identify these protein bands. The N-terminal sequences of 23 kDa and 24 kDa proteins were found to be ‘TCSPQPGG’, whereas the N-terminal amino acid sequence of the protein of 18 kDa molecular weight was ‘ISSCNGPP’. A homology search using BLAST for proteins with matched N-terminal sequences was performed, and all the three samples matched with the N-terminal proximal sequence of kiwellin (Act d 5), a cysteine-rich cell wall-related protein (Fig. 9).

## Discussion

Kiwifruit is a popular fruit of the genus *Actinidia* that contains abundant bioactive substances such as dietary fiber, carbohydrates, natural sugars, vitamins, minerals, and antioxidants, and hence, globally consumed (11). However, many food allergy cases have also been reported to be caused by kiwifruit (13). Whether kiwifruit proteins can transdermally sensitize a mouse model has not been fully investigated. Therefore, in this study, we demonstrated transdermal sensitization to kiwifruit proteins in a mouse model.

ELISA showed significantly high levels of both kiwifruit-specific IgE and IgG1 antibodies in both the kiwifruit groups. Immunoblotting revealed several major IgE- and IgG1-binding protein bands of around 18 and 20–25 kDa. Cross-reactivity was observed between the two varieties of kiwifruits. Therefore, the protein bands detected could be transdermal sensitization antigens found in kiwifruits. In addition, no significant difference was found between the IgE and IgG1 levels produced between green and gold kiwifruits, and therefore, no difference in the transdermal sensitization ability between these varieties of kiwifruits was confirmed.

Using green kiwifruit-specific IgG1 binding as an indicator, three transdermal sensitizing allergens of 18, 23, and 24 kDa molecular weights were purified and identified, and their partial amino acid sequence was found to be similar to that of kiwellin. Among them, the 18-kDa sample was considered to be a degradation product of kiwellin called KiTH (26). The purified and identified transdermal sensitizing allergens of 23 and 24 kDa were identical to the N-terminal amino acid sequence of kiwellin. The differences between the two molecules might be because of post-translational modifications or limited proteolysis around the C-terminal region of the proteins; however, the details of the molecular weight differences between the two allergens are unknown.

In the past three decades, the incidence of kiwifruit allergy due to actinidin, kiwellin and thaumatin-like protein has increased (27). The function of kiwellin, a putative transdermal sensitizing allergen, is unclear; however, this cysteine-rich protein is one of the major proteins present in large quantities in the edible parts of the kiwifruit (28). Kiwellin acts as a cell wall or a plant defense protein to offset foreign effector activities with high specificity (29). A recent study using transcriptomic data showed that the level of kiwellin homolog was strongly increased in potatoes infected with the oomycete of the potato pest fungus (30). Increased expression of the gene encoding kiwellin has also been observed in adult whitefly infected chrysanthemum. Based on this evidence, kiwellin may be a multifaceted broad scaffold protein that counteracts secreted pathogen effectors (29). Interestingly, many plant allergens are known to be biodefense-related proteins belonging to the pathogenesis-related protein family. Kiwellin, which we found as a transdermal sensitizing antigen, may also be categorized as a class of biodefense-related proteins based on their properties. Why such biodefense-related proteins are prone to be allergenic is unknown but plant–animal interactions might be a major factor involved in it.

Kiwellin is a protein consisting of two domains, an N-terminal kissper (residues 1–39) that consists of six cysteines and an N-terminal 4 kDa peptide moiety, and a C-terminal domain that consists of eight cysteines (residues 40–189) (28). Some of the sera specific for kiwellin from green kiwi were more responsive to KiTH than to kiwellin. On the other hand, some sera responded more highly to kiwellin than to KiTH. Thus, kiwellin might have a hidden IgE-binding epitope that becomes available in KiTH, following the removal of kissper, and kissper might be an IgE-binding epitope by itself (26). In ripe green kiwifruit, kiwellin is degraded by the cysteine protease actinidin, generating a 4-kDa N-terminal domain kispper and a 16-kDa C-terminal domain KiTH (31). Actinidine is also abundant in kiwifruit and accounts for more than 50% of the mature kiwifruit protein (32). Because of these properties, actinidine is known as the major allergen in kiwifruit as act d1 (33). However, in the present study, kiwellin caused IgE and IgG1 production more clearly than actinidin. Therefore, kiwellin might be more sensitizing than actinidin as a transdermal sensitizing antigen in kiwifruit.

## Conclusions

In this study, we showed that both green and gold kiwifruit have transdermal sensitization potential in a murine model system, and identified kiwellin as a new biodefense protein as a sensitization antigen. This molecule is already known as a minor allergen of kiwifruit for humans but could be a major allergen in transdermal sensitization. These findings provide new targets for strategies to reduce the risk of allergy caused by kiwifruit.
